# Integrated Lipidomics and Metabolomics Study of Four Chemically Induced Mouse Models of Acute Intrahepatic Cholestasis

**DOI:** 10.3389/fphar.2022.907271

**Published:** 2022-06-08

**Authors:** Weiwei Li, Hui Chen, Yihan Qian, Shouchuan Wang, Zichen Luo, Jinjun Shan, Xiaoni Kong, Yueqiu Gao

**Affiliations:** ^1^ Department of Formulaology, School of Basic Medicine Science, Shanghai University of Traditional Chinese Medicine, Shanghai, China; ^2^ Central Laboratory, ShuGuang Hospital Affiliated to Shanghai University of Traditional Chinese Medicine, Shanghai, China; ^3^ Jiangsu Key Laboratory of Pediatric Respiratory Disease, Institute of Pediatrics, Affiliated Hospital of Nanjing University of Chinese Medicine, Nanjing, China

**Keywords:** lipidomics, metabolomics, acute intrahepatic cholestasis, mouse model, LC-MS, GC-MS

## Abstract

Lithocholic acid (LCA), alpha-naphthyl isothiocyanate (ANIT), 3,5-diethoxycarbonyl-1,4-dihydrocollidine (DDC), and ethinyl estradiol (EE) are four commonly used chemicals for the construction of acute intrahepatic cholestasis. In order to better understand the mechanisms of acute cholestasis caused by these chemicals, the metabolic characteristics of each model were summarized using lipidomics and metabolomics techniques. The results showed that the bile acid profile was altered in all models. The lipid metabolism phenotype of the LCA group was most similar to that of primary biliary cirrhosis (PBC) patients. The ANIT group and the DDC group had similar metabolic disorder characteristics, which were speculated to be related to hepatocyte necrosis and inflammatory pathway activation. The metabolic profile of the EE group was different from other models, suggesting that estrogen-induced cholestasis had its special mechanism. Ceramide and acylcarnitine accumulation was observed in all model groups, indicating that acute cholestasis was closely related to mitochondrial dysfunction. With a deeper understanding of the mechanism of acute intrahepatic cholestasis, this study also provided a reference for the selection of appropriate chemicals for cholestatic liver disease models.

## Introduction

Cholestasis is the impairment of bile formation and flow. Intrahepatic cholestasis may result from defective bile secretion from hepatocytes and/or bile duct cells or mechanical obstruction of bile flow through intrahepatic bile ducts (Beuers et al., 2015). The causes of intrahepatic cholestasis are complex, such as toxins/drugs, cholangitis, local tumors, genetic alterations, autoimmune disorders, and pregnancy (Kim et al., 2000; Onofrio and Hirschfield, 2020).

Notably, although jaundice is the hallmark symptom of cholestasis, patients often have no obvious clinical manifestations in the early stage of the disease. It is well known that the liver has a strong compensatory regeneration ability after parenchymal injury or cell loss, as well as a variety of adaptive and protective mechanisms for cholestasis, which may be the underlying reason for the lack of obvious clinical manifestations in the early stage of cholestatic liver disease. Patients may progress “silently” for years prior to the development of symptoms such as jaundice and pruritus. At this time, cholestatic liver diseases often lead to liver failure and cirrhosis, requiring liver transplantation (Padda et al., 2011). Therefore, we need to pay more attention to the acute stage of intrahepatic cholestasis, on the one hand, to find biomarkers that can characterize the acute stage of intrahepatic cholestasis, and on the other hand, to clarify the pathophysiological process of the acute stage of intrahepatic cholestasis.

Lithocholic acid (LCA), alpha-naphthyl isothiocyanate (ANIT), 3,5-diethoxycarbonyl-1,4-dihydrocollidine (DDC), and ethinyl estradiol (EE) are four commonly used chemicals for the construction of intrahepatic cholestasis (Tjandra et al., 2000; Staudinger et al., 2001; Marrone et al., 2016; Yang et al., 2017; Slijepcevic et al., 2018; Tardelli et al., 2020). LCA is a hydrophobic secondary bile acid formed by bacterial 7α-dehydroxylation of chenodeoxycholic acid (CDCA) and ursodeoxycholic acid (UDCA) in intestine (Zeng et al., 2016). Mice fed with LCA led to development of segmental bile duct obstruction and destructive cholangitis (Fickert et al., 2006). ANIT is metabolized by hepatocytes and secreted as a glutathione conjugate via MRP2 into bile (Carpenter-Deyo et al., 1991). This conjugate, however, is unstable and bile ducts are exposed to high concentrations of unconjugated, toxic ANIT, resulting in bile duct epithelial cells and hepatocyte damage and necrosis. Administration of DDC can results in increased biliary secretion of porphyrins with subsequent formation of protoporphyrin plugs, obstructing small bile ducts and causing cholestasis (Fickert et al., 2007). EE-induced intrahepatic cholestasis is a rodent model widely used to investigate the molecular mechanisms of estrogen-induced bile secretion dysfunction.

Due to the various causes of acute cholestatic liver disease, selecting appropriate chemicals for modeling remains a problem worth of exploring. For example, primary cholestatic liver diseases, such as primary biliary cirrhosis (PBC) and primary sclerosing cholangitis (PSC), have different pathogenic mechanisms, even though the pathological manifestations of these two diseases are both bile duct necrotizing inflammatory injury (Jansen et al., 2017). Studies have shown that PBC and PSC have different bile acid disorders and metabolic characteristics (Trottier et al., 2012; Bell et al., 2015). Therefore, it seems inappropriate to use the same model, such as the ANIT-induced cholestasis model, to characterize both PBC and PSC (Tjandra et al., 2000). Reviewing studies on cholestatic liver disease models, it has been shown that different models induced by ANIT, DDC and LCA have their own metabolic characteristics. The level of plasma phospholipids was elevated in ANIT and DDC models, whereas that was depleted in LCA model. Besides, the level of protoporphyrin IX was significantly increased in the liver of DDC model (Yang et al., 2018). Therefore, the use of ANIT, DDC, LCA, and other chemically induced mouse models to characterize the same disease, such as PSC, is also questionable (Fickert et al., 2014).

Mass spectrometry (MS)-based omics techniques are now widely used to profile small molecules to confer comprehensive snapshots of the metabolic phenotypes of diseases. Metabolomics mainly focuses on polar metabolites, while lipidomics strives to comprehensively identify and quantify all kinds of lipid molecular species (Wang et al., 2020). Combining non-targeted metabolomics and lipidomics, the data obtained can comprehensively characterize the metabolic disorders in the disease process and help understand disease mechanisms. Lipidomics is widely used in the study of liver diseases, such as nonalcoholic steatohepatitis (Musso et al., 2018), hepatocellular carcinoma (Sangineto et al., 2020), liver cirrhosis (Lopez-Vicario et al., 2020) and so on. Previous studies on cholestatic liver disease mostly focused on the disorder of bile acid metabolic pathway. However, recent studies have gradually discovered that besides their well-characterized functions in cholesterol homeostasis and nutrient absorption, bile acids are also important metabolic regulators. They participate in the whole process of lipid metabolism by activating specific nuclear receptors, G-protein-coupled receptors, and multiple signal pathways (Kwong et al., 2015). For example, clinical studies have found that the disease process of PBC and PSC involves both bile acid and lipid metabolism disorders (Bell et al., 2015). Lipid metabolism disorders may be closely related to the pathogenesis of cholestatic liver disease. Recently, lysophosphatidylcholine (LysoPC) has been discovered as a novel cholestatic pruritogen (Chen et al., 2021).

In order to further summarize the similarities and differences of acute intrahepatic cholestasis models caused by different chemicals, and to clarify the clinical types of cholestatic liver disease represented by each model, we used ANIT, DDC, LCA, and EE to construct four different acute intrahepatic cholestasis mouse models. We used the combined technique of lipidomics and metabolomics to comprehensively analyze the metabolic profile of liver tissue samples of each model. The metabolic pathway disorders involved in each model and the pathogenesis of each model were also summarized.

## Materials and Methods

### Materials and Chemicals

Methanol and acetonitrile (ACN) were obtained from Merck KGaA (Darmstadt, Germany). N,O-Bis (trimethylsilyl) trifluoroacetamide (BSTFA) with 1% trimethylchlorosilane (TMCS), alpha-naphthyl isothiocyanate (ANIT), ethinyl estradiol (EE), 3,5-diethoxycarbonyl-1,4-dihydrocollidine (DDC), lithocholic acid (LCA), corn oil, methoxyamine hydrochloride, pyridine, a standard n-alkanes mixture (C_8_–C_40_), and 1,2–^13^C_2_-myristic acid were purchased from Sigma-Aldrich (St. Louis, United States). Methyl tert-butyl ether (MTBE), isopropanol, formic acid, and ammonium formate were obtained from ROE Scientific (St. Louis, United States). Internal standard lysophosphatidylethanolamine (LysoPE) (17:1), sphingomyelin (SM) (17:0), and phosphatidylethanolamine (PE) (17:0/17:0) were purchased from Avanti Polar Lipids (Alabaster, United States).

Reference standards LCA, tauroursodeoxycholic acid (TUDCA), taurochenodeoxycholic acid (TCDCA), and taurodeoxycholic acid (TDCA) were purchased from Yuanye Biotechnology Co., Ltd. (Shanghai, China). Taurohyodeoxycholic acid (THDCA) was purchased from Pufeide Biotechnology Co., Ltd. (Chengdu, China). Deoxycholic acid (DCA), glycocholic acid (GCA), and UDCA were purchased from Aladdin Biochemical Technology Co., Ltd. (Shanghai, China). Hyodeoxycholic acid (HDCA) and taurocholic acid (TCA) were purchased from McLean Biochemical Technology Co., Ltd. (Shanghai, China). Cholic acid (CA) and CDCA were purchased from Siyu Chemical Technology Co., Ltd. (Shanghai, China). Tauro-beta-muricholic acid (T-beta-MCA), alpha-muricholic acid (alpha-MCA), beta-muricholic acid (beta-MCA), omega-muricholic acid (omega-MCA), and d9-taurochenodeoxycholic acid (d9-TCDCA) were purchased from Isoreag Co., Ltd. (Shanghai, China).

### Establishment of Acute Intrahepatic Cholestasis Models

Sixty four male C57BL/6J mice, aged 4–6 weeks (20 ± 2 g) were purchased from Jiangsu Jicui Yaokang Biotechnology Co., Ltd. (license number: SCXK (Jiangsu) 2018-0008). All mice were acclimated for 1 week before the experiment and were kept under the standard conditions with 12 h light dark cycle at a temperature of 23–24°C, a humidity of 50–60%, with free access to food and water.

**FIGURE 1:**
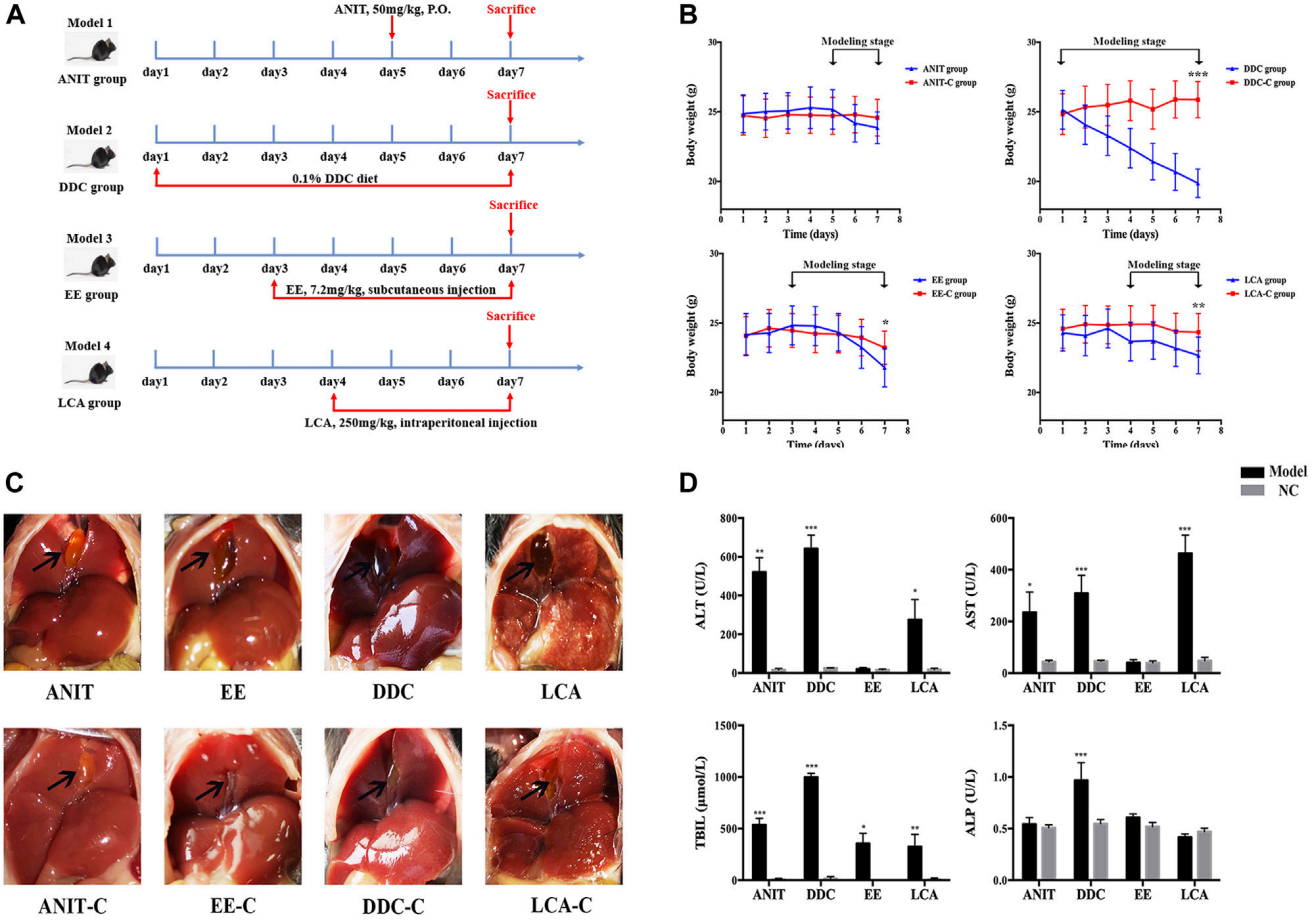
Acute intrahepatic cholestasis murine models induced by ANIT, DDC, EE and LCA. **(A)** Experimental protocols to establish the four models. **(B)** The body weight of mice in each group were evaluated daily. **(C)** Photographs of representative livers. Gall bladders are marked by arrows. **(D)** The data of serum ALT, AST, TBIL and ALP indicate liver injury and cholestasis in all four murine models. Data are the mean ± S.D. (n = 8). *p < 0.05 versus Control, **p < 0.01 versus Control, ***p < 0.001 versus Control.

After 7 days of adapting, mice were randomly divided into 8 groups with 8 mice in each group: 1) ANIT; 2) control of ANIT (ANIT-C); 3) EE; 4) control of EE (EE-C); 5) DDC; 6) control of DDC (DDC-C); 7) LCA; 8) control of LCA (LCA-C). Due to the different modeling days required by each model, in order to ensure that each model group completed modeling at the same time, the modeling start time of each model was slightly different, as shown in Figure 1A. From day 1 to day 4, the ANIT and ANIT-C group have no special treatment. On the fifth day after fasted overnight, ANIT dissolved in corn oil was given at a single dose of 50 mg/kg by gavage for ANIT group, the ANIT-C group was given the same dose of corn oil (Zhang et al., 2018). From day 1 to day 2, the EE and EE-C group have no special treatment. EE (7.2 mg/kg body weight) was administered subcutaneously for the next 5 consecutive days, the EE-C group received only the EE vehicle (propylene glycol) (Marrone et al., 2016). From day 1 to day 7, the DDC group was placed on a chow diet supplemented with 0.1% DDC (Slijepcevic et al., 2018). The DDC-C group fed normal chow diet without DDC. From day 1 to day 3, the LCA and LCA-C group have no special treatment. The LCA group received a daily intraperitoneal (i.p.) injection of LCA (0.125 mg/kg in corn oil) twice a day from day 4 to day 7 and sacrificed 24 h after the last dose of LCA. The LCA-C group was given the same dose of corn oil (Staudinger et al., 2001).

Mice were sacrificed on the 7th day after fasting for 12 h. Blood samples and liver tissues were collected for further studies. Serum samples were separated by centrifuging at 3000 rpm for 10 min at 4°C. In order to reduce the intra group differences caused by the selection of liver samples from different parts, the right middle lobe of each mouse’s liver was uniformly fixed in 10% neutral buffered formalin for histopathological examination. The left lateral lobe of the liver (the largest part of the liver lobe) of every mouse was roughly divided into four parts, and immediately frozen in liquid nitrogen and kept at −80°C until use for lipidomics analysis, GC-MS based metabolomics analysis, bile acid quantification and qPCR detection. The animal care procedures and experimental protocols were approved by Animal Research Ethics, Affiliated Hospital of Nanjing University of Chinese Medicine (2020 DW-37-02).

### Serum Biochemistry

Serum alanine aminotransferase (ALT), aspartate aminotransferase (AST), total bilirubin (TBIL), and alkaline phosphatase (ALP) levels were measured by commercial test kits from Jiancheng Bioengineering Institute (Nanjing, China) (cat. no. C009-2-1, C010-2-1, C019-1-1, A059-2-2).

### Histopathological Examination

Liver samples for histopathological examination were fixed in 10% neutral buffered formalin, embedded in paraffin, sectioned at a 5 μM thickness, and stained with Hematoxylin and Eosin (H&E) for necrosis and other structural changes.

### Quantification of Bile Acids

Liver sample (50 mg) was spiked with 200 μL water and homogenized for 3 min, then the liver tissue homogenate was centrifuged at 13,200 rpm for 10 min at 4°C. 100 μL supernatant was transferred to a new tube. The remaining supernatant in the previous tube was absorbed and discarded, then 200 μL methanol was added and homogenized again for 3 min. Next, the liver tissue homogenate was centrifuged at 13,200 rpm for 10 min at 4°C. 150 μL supernatant was mixed with the previous supernatant. The mixture was vortexed for 3 min and centrifuged at 18,000 rpm for 10 min at 4°C. 40 μL supernatant was transferred to a new tube and mixed with 170 μL methanol containing 10 ng d9-TCDCA as an internal standard. The mixture was vortexed for 30 s and centrifuged at 18,000 rpm for 10 min at 4°C. Finally, the supernatant was transferred to an autosampler plate for analysis. The injection volume of each sample was 2 μL.

The chromatography experiment was carried out using a UHPLC system (Thermo Fisher, United States), equipped with an ACQUITY UPLC BEH C18 column (100 mm × 2.1 mm, 1.7 μm). Mass spectrometry detection was operated on TSQ Vantage triple-stage quadrupole mass spectrometer (Thermo Fisher, United States) equipped with an electrospray ionization (ESI) source. Quantification of the analytes was performed using selective reaction monitor (SRM) in the negative ionization mode. Column temperature was 45°C. Mobile phase A was water, and mobile phase B was ACN: methanol (95: 5), both containing 0.1% formic acid with the flow rate of 0.4 ml/min. The gradient program was as follows: 0 min, 26% B, 2 min, 26% B, 4 min, 30% B, 5 min, 35% B, 18 min, 60% B, 21 min, 100% B, 22 min, 26% B, 25 min, 26% B.

The spray voltage was set to 3.5 kV. Capillary and auxiliary gas heater temperatures were set at 320 and 400°C respectively. Nitrogen was used as both the sheath gas at a flow rate of 45 arbitrary units and the auxiliary gas at a flow rate of 25 arbitrary units.

### Quantitative Real-Time PCR

Total hepatic RNA from mouse liver tissues was extracted using a tissue total RNA isolation kit according to the manufacturer’s instruction (Vazyme, CHN). Purification of 500 ng RNA and reverse transcription to cDNA by reverse transcription PCR was performed using a HiScript II Q RT SuperMix for qPCR (Vazyme, CHN). Quantitative real-time PCR was performed using a ChamQ Universal SYBR qPCR Master Mix (Vazyme, CHN). The reaction conditions were as follows: 95°C for 30 s, followed by 40 cycles of denaturation at 95°C for 10 s and extension at 60°C for 30 s. The primers were shown in Table 1. The results were analyzed using the QuantStudio 7 Flex detection system (Applied Biosystems Co., United States) and the 2−ΔΔCT method to assess the levels of mRNAs encoding targeted genes normalized to GAPDH.

**TABLE 1:** Primer sequences.

No	Gene name	Forward (5′->3′)	Reverse (5′->3′)
1	Fxr	GGC​AGA​ATC​TGG​ATT​TGG​AAT​CG	GCC​CAG​GTT​GGA​ATA​GTA​AGA​CG
2	Fgf15	ACG​TCC​TTG​ATG​GCA​ATC​G	GAG​GAC​CAA​AAC​GAA​CGA​AAT​T
3	Fgfr4	CTG​ACT​CGC​AGA​CGA​CAT​GAG	AGG​CCA​TGA​TCT​TGA​GAT​GAG​A
4	Cyp7a1	CAA​GAA​CCT​GTA​CAT​GAG​GGA​C	CAC​TTC​TTC​AGA​GGC​TGC​TTT​C
5	Cyp8b1	ATC​GCC​TGA​AGC​CCG​TGC​AG	AGC​TGG​GGA​GAG​GAA​GGA​GTG​C
6	Cyp27a1	CCT​CAC​CTA​TGG​GAT​CTT​CAT​C	TTT​AAG​GCA​TCC​GTG​TAG​AGC
7	Bsep	CAA​TGT​TCA​GTT​CCT​CCG​TTC​A	TCT​CTT​TGG​TGT​TGT​CCC​CAT​A
8	Mrp3	ATC​ACA​GCC​AGT​TCA​AAG​CCA	GGC​AGG​AGC​AAG​GTC​TCT​TAA​A
9	Ntcp	ACT​GGC​TTC​CTG​ATG​GGC​TAC	GAG​TTG​GAC​GTT​TTG​GAA​TCC​T
10	Mdr2	CGG​CGA​CTT​TGA​ACT​AGG​CA	CAG​AGT​ATC​GGA​ACA​GTG​TCA​AC
11	GAPDH	TCC​ACC​ACC​CTG​TTG​CTG​TAG	GAC​CAC​AGT​CCA​TGA​CAT​CAC​T

### UHPLC-Q-Exactive-MS Based Lipidomics Analysis

#### Liver Sample Preparation

To perform a comprehensive lipidomic profiling, we developed a sample preparation strategy for covering different classes of lipids based on the liquid–liquid MTBE extraction (Yang et al., 2018). Briefly, liver sample (20 mg) was spiked with 200 μL deionized water and homogenized for 15 min, then 20 μL liver tissue homogenate was mixed with 225 μL ice-cold methanol containing LysoPE (17:1), SM (17:0) as internal standard for positive ion mode and PE (17:0/17:0) as internal standard for negative ion mode (internal standard concentration is about 5 μg/ml). The mixture was vortexed for 10 s, then 750 μL MTBE was added, and the mixture was shaken for 10 min at 4°C. After adding 188 μL of deionized water, the mixture was vortexed for 20 s and then centrifuged at 18,000 rpm for 2 min at 4°C. Three hundred and 50 μL of supernatant were transferred to fresh tubes and dried in a SpeedVac sample concentrator at 45°C for 2 h. The dried aliquots were reconstituted with 110 μL methanol: toluene (9:1), vortexed for 10 min, and shaken for 10 min at 4°C, and then centrifuged at 18,000 rpm for 10 min at 4°C. Finally, the supernatant was transferred to an autosampler plate for LC-MS analysis.

### Untargeted Lipidomic Analysis

A Dionex UltiMate 3000 ultra-high performance liquid chromatography (UHPLC) system (Santa Clara, CA, United States) coupled online via an electrospray ionization source (ESI) with a Q ExactiveTMMS instrument (Thermo Fisher Scientific, United States) was applied for the untargeted lipidomic analysis.

To detect lipids, 2 μL aliquots of sample solution, maintained at 4°C in an auto sampler, was injected onto a reversed phase Waters Acquity UPLC CSH C18 (100 mm × 2.1 mm, 1.7 μm) maintained at 60°C by gradient elution. Mobile phase A was water: ACN (6:4), and mobile phase B was isopropanol: ACN (9: 1), both containing 10 mM ammonium formate and 0.1% formic acid. The flow rate was 0.3 ml/min, with the elution gradient as follows: 0–2.0 min, 15–30% B; 2.0–2.5 min, 30–48% B; 2.5–11.0 min, 48–82% B; 11.0–11.5 min, 82–99% B; 11.5–12 min, 99% B; 12–13 min, 99–15% B; and 13.0–15.0 min, 15% B.

All MS experiments were performed in positive and negative ion modes using a heated electrospray ionization source. The source and ion transfer parameters applied were as follows: spray voltage 3.5 kV (positive) and 3.0 kV (negative). For both modes, the sheath gas, aux gas, capillary temperature and heater temperature were maintained at 45, 10 (arbitrary units), 325 and 300°C, respectively. The S-Lens RF level was set at 50. The Orbitrap mass analyzer was operated at a resolving power of 60,000 in full-scan mode (scan range: 215–1800 m/z).

### GC-MS Based Metabolomics Analysis

Liver sample (50 mg) was spiked with 1 ml methanol and homogenized for 10 min, then 600 μL liver tissue homogenate was transferred to a new tube and centrifuged at 14,000 rpm for 10 min at 4°C. Two hundred and 80 μL of supernatant was transferred to a new tube and mixed with 20 μL methanol containing 6 μg 1,2–^13^C_2_-myristic acid as an internal standard. The mixture was vortexed for 3 min and rapid centrifuged, then the mixture was dried in a SpeedVac sample concentrator at 45°C for 2 h. The dried aliquots were combined with 30 μL methoxyamine hydrochloride in pyridine (10 mg/ml), then vortexed for 5 min and shaken at 30°C for 90 min using the Thermo Mixer C (Eppendorf, Germany). 30 ml of BSTFA containing 1% TMCS were added to the sample, which was then shaken at 37°C for 30 min. The mixture was then transferred to a sampler vial with a glass insert and subjected to GC–MS analysis. The injection volume of each sample was 1 μL.

As described previously, analysis was performed on a TRACE 1310 gas chromatograph equipped with an AS 1310 autosampler connected to a TSQ 8000 triple quadrupole mass spectrometer (Thermo Fisher Scientific, United States) (Li et al., 2021; Li et al., 2021). A TG-5MS GC column (Thermo, 0.25 mm, 30 m) in split mode with a 20:1 ratio of the above samples was used. Helium was used as the carrier gas, and was maintained at a constant flow of 1.2 ml/min. The oven temperature was initially maintained at 60°C for 1 min, then increased to 320°C at 20°C/min, and then held constant for 5 min. The transfer line temperature between the gas chromatograph and the mass spectrometer was set to 250°C. Electron impact ionization at 70 eV was employed, with an ion source temperature of 280°C. Mass spectra were acquired with a scan range of 50–500 m/z and a time range of 3.5–19 min. To detect and eliminate retention time shifts, the standard n-alkane mixture (C_8_–C_40_) was injected into the GC–MS during analysis of each batch of samples.

### Data Processing and Statistical Analysis

LC-MS and GC-MS raw data acquired from Xcalibur 2.2 software (Thermo Fisher, United States) were converted to the “abf” format with the ABF converter (http://www.reifycs.com/AbfConverter/ index.html). For data processing, the MS-DIAL (Tsugawa et al., 2015) software program was used for raw peak exaction, data baseline filtering and calibration, peak alignment, deconvolution analysis, and peak identification.

For lipid identification, accurate mass and MS/MS matching was used with the public LipidBlast library of over 200,000 MS/MS spectra (Kind et al., 2013). As shown in Supplementary Figure S1, we gave some examples of the identification process of lipids. The experimental mass spectra (upper panel) were matched against mass spectrometry libraries LipiBlast (lower panel). The compound identification was performed by the weighted similarity score of retention time, accurate mass, isotope ratio, and MS/MS spectra.

For GC-MS metabolites identification, spectra were matched against the FiehnLib mass spectral and retention index library (Kind et al., 2009). In addition, metabolites were also identified through matching of their mass spectra against the reference spectra in the NIST 2014 standard database built-in Xcalibur 2.2 software. Briefly, metabolites were identified by comparing the mass fragments with the NIST 2014 standard database with a similarity of >70% and finally verified using the available reference standards.

As described previously, data normalization was completed using MetaboAnalyst 5.0 (http://www.metaboanalyst.ca) (Li et al., 2021). Multivariate statistical analysis of PCA and PLS-DA, volcano plot and Student’s t test were also performed using MetaboAnalyst 5.0. Significant metabolites were selected from the volcano plot with fold change (FC) > 2 or <0.5, with a Student’s t test *p* value threshold <0.05, and a false discovery rate (FDR) < 0.05 in the above PLS-DA model.

Other experimental data were represented as means ± standard deviation (SD). Statistical differences between two groups were determined using a Two-tailed Student’s t-test. All statistical analyses were performed in GraphPad Prism 6.0 (GraphPad Software Inc., United States). Results were considered to be statistically significant with *p* < 0.05.

## Results

### Characteristics of Four Acute Intrahepatic Cholestasis Models

During the modeling process, all model groups had different degrees of weight loss. After modeling, the weight of DDC group, EE group and LCA group significantly decreased compared with the normal control (NC) group (Figure 1B). Morphological examination revealed gallbladder enlargement in all model groups. The liver of the DDC group was darker, while the liver necrosis of the LCA group was obvious (Figure 1C). Serum biochemical indicators of hepatocyte injury and cholestasis were also determined (Figure 1D). The serum ALT and AST levels were significantly increased in the ANIT, DDC, and LCA group. TBIL levels were significantly increased in all four model groups. ALP was significantly increased only in DDC group.

**FIGURE 2:**
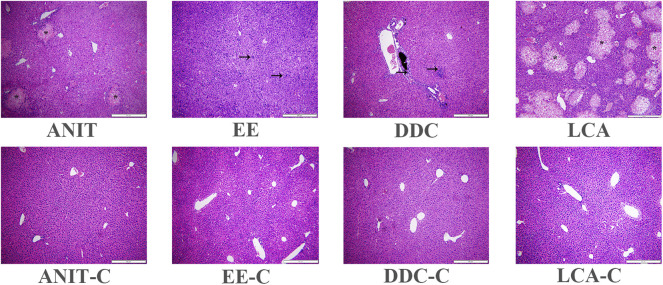
Representative H&E stained liver sections (10 × 10). Characteristic bile infarcts as shown by the asterisk, inflammatory infiltration, destructive interlobular ducts as shown by the arrow.

Histopathological examination showed that the liver tissue of all the control groups exhibited a normal structure without abnormal morphological changes, while all the model groups showed acute infiltration by polymorphonuclear neutrophils. The ANIT and the LCA group also showed edema, sinusoid congestion, and hepatic necrosis. Significant cholestasis in intrahepatic bile ducts was also observed in the DDC group (Figure 2).

### Quantitative Detection Results of Bile Acids in Each Model Group

**FIGURE 3:**
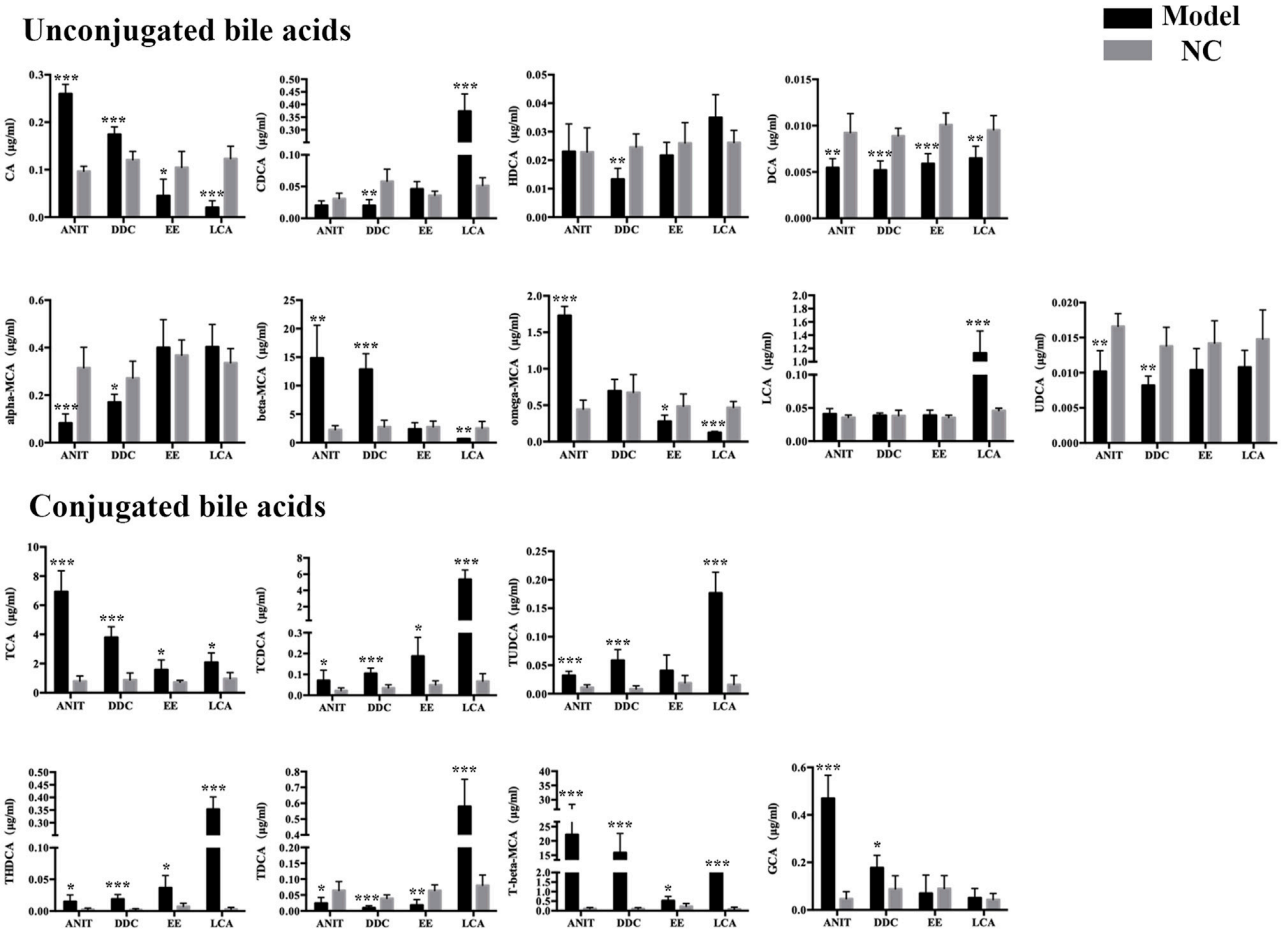
ANIT, DDC, EE and LCA-induced disordered bile acid homeostasis in mice. Data are the mean ± S.D. (n = 5).*p < 0.05 versus Control, **p < 0.01 versus Control, ***p < 0.001 versus Control.

Liver bile acid levels of all the groups were measured by UPLC-MS/MS. As shown in Figure 3, compared with the NC group, the bile acid homeostasis of all model groups was imbalanced. The DDC group and the ANIT group had similar bile acid disorder characteristics. The levels of unconjugated bile acids CA, beta-MCA, most conjugated bile acids, such as TCA, TCDCA, T-beta-MCA, TUDCA, THDCA, GCA, were increased, and the levels of unconjugated bile acids alpha-MCA, UDCA, CDCA, DCA, and conjugated bile acid TDCA were decreased in the DDC group and the ANIT group. In the EE group, unconjugated bile acids CA, omega-MCA, and DCA were decreased, most taurine-conjugated bile acids, such as TCA, TCDCA, TUDCA, THDCA and T-beta-MCA, were increased. In the LCA group, most unconjugated bile acids were decreased, and the significant increase in LCA was associated with the use of LCA as the modeling drug. Similar to other model groups, conjugated bile acids were increased in the LCA group.

**FIGURE 4:**
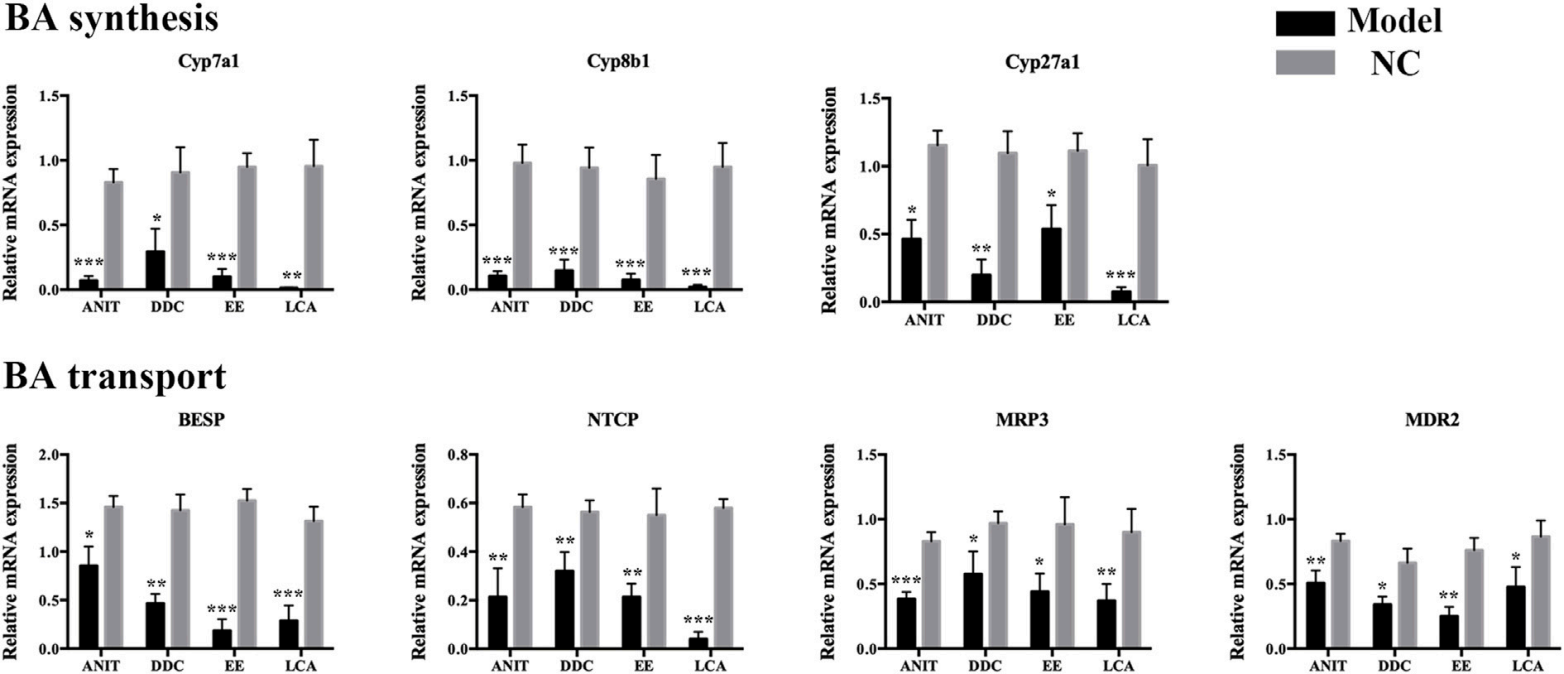
The mRNA expression of BA synthetic enzymes and transporters in livers of the four models. Data are the mean ± S.D. (n = 5).*p < 0.05 versus Control, **p < 0.01 versus Control, ***p < 0.001 versus Control.

To further clarify the mechanism of bile acid homeostasis imbalance in each model group, the expression of bile acid synthetic enzymes and transporters in the liver were also determined. For enzymes, Cyp7a1, Cyp8b1, and Cyp27a1 levels were down-regulated in four model groups. For transporters, bile salt export pump (BSEP), Na^+^-taurocholate cotransporting peptide (NTCP), multidrug resistance associated protein 3 (MRP3), and multidrug resistance protein 2 (Mdr2) levels were also down-regulated in four model groups (Figure 4). The results indicated that the bile acid synthesis and transport in each model were obstructed.

### Identification of Differential Lipids Involved in Each Model

**FIGURE 5:**
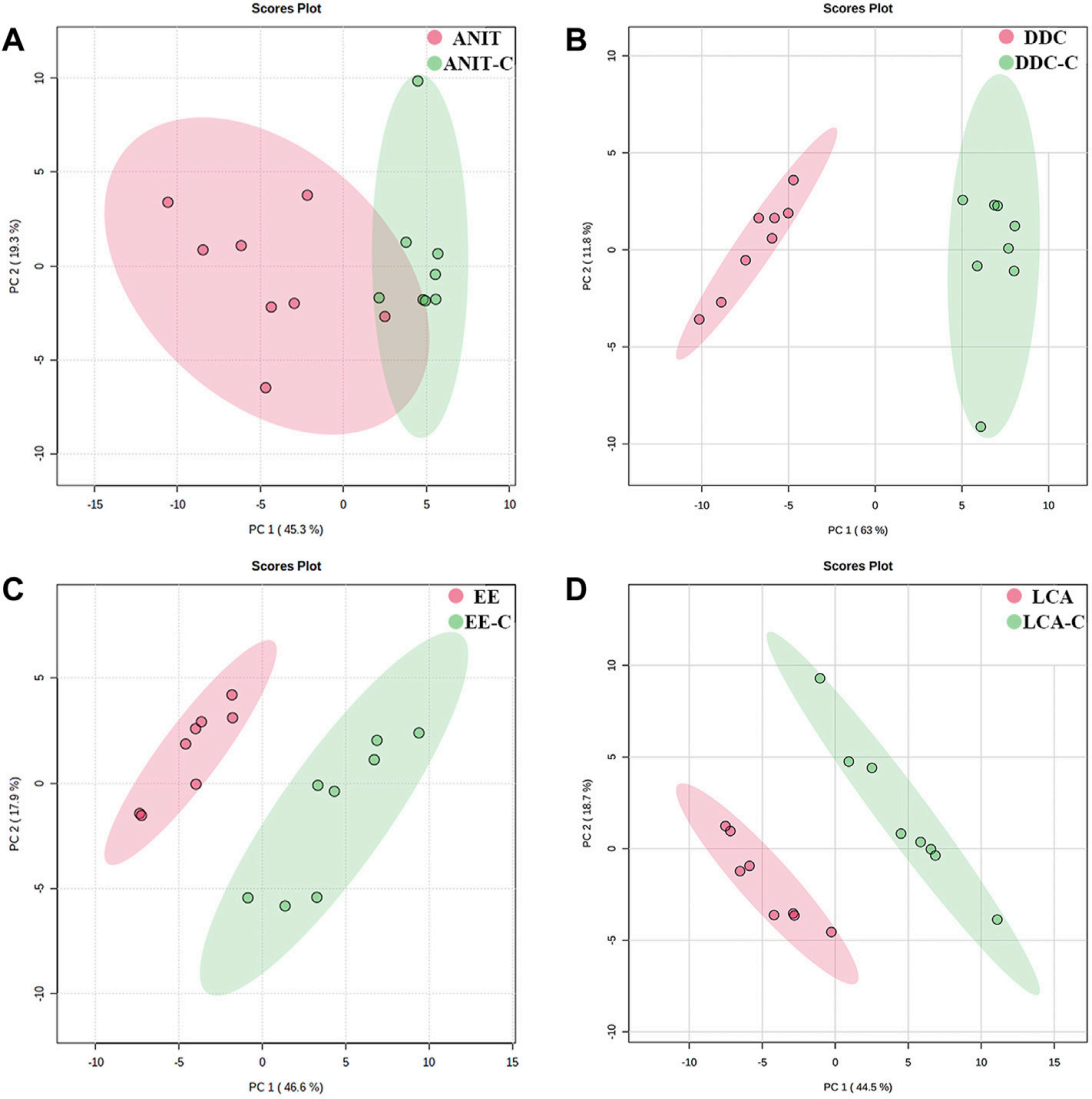
PCA score plots based on the lipid profiling of the four models versus their NC groups. **(A)** ANIT group vs. ANIT-C group. **(B)** DDC group vs. DDC-C group. **(C)** EE group vs. EE-C group. **(D)** LCA group vs. LCA-C group. (n = 8).

The lipid profiles of liver samples were investigated by UHPLC-Q-Exactive-MS. After alignment and normalization of the data sets, we obtained a total of 490 known lipids (305 in the positive mode and 185 in the negative mode). Subsequently, unsupervised principal component analysis (PCA) was performed to find clustering trends of lipid profiles among the four groups. As shown in Figure 5, the ANIT, DDC, EE, and LCA groups showed a separation trend with their NC groups. Supervised PLS-DA models were constructed to further evaluate differences between the models versus their NC groups. As shown in Supplementary Figure S2, the model groups were significantly separated from the NC groups. Besides, as shown in Supplementary Figure S3, the DDC and the LCA group were significantly separated from the other groups. The ANIT and the DDC group were clustered together, suggesting that the lipid metabolism profiles of the two cholestatic models were similar. The reproducibility of data was assessed by using quality control (QC) samples. As shown in Supplementary Figure S4, the PCA scores plot revealed a clear cluster of the QC samples, indicating the high stability and reproducibility of the instrument.

**FIGURE 6:**
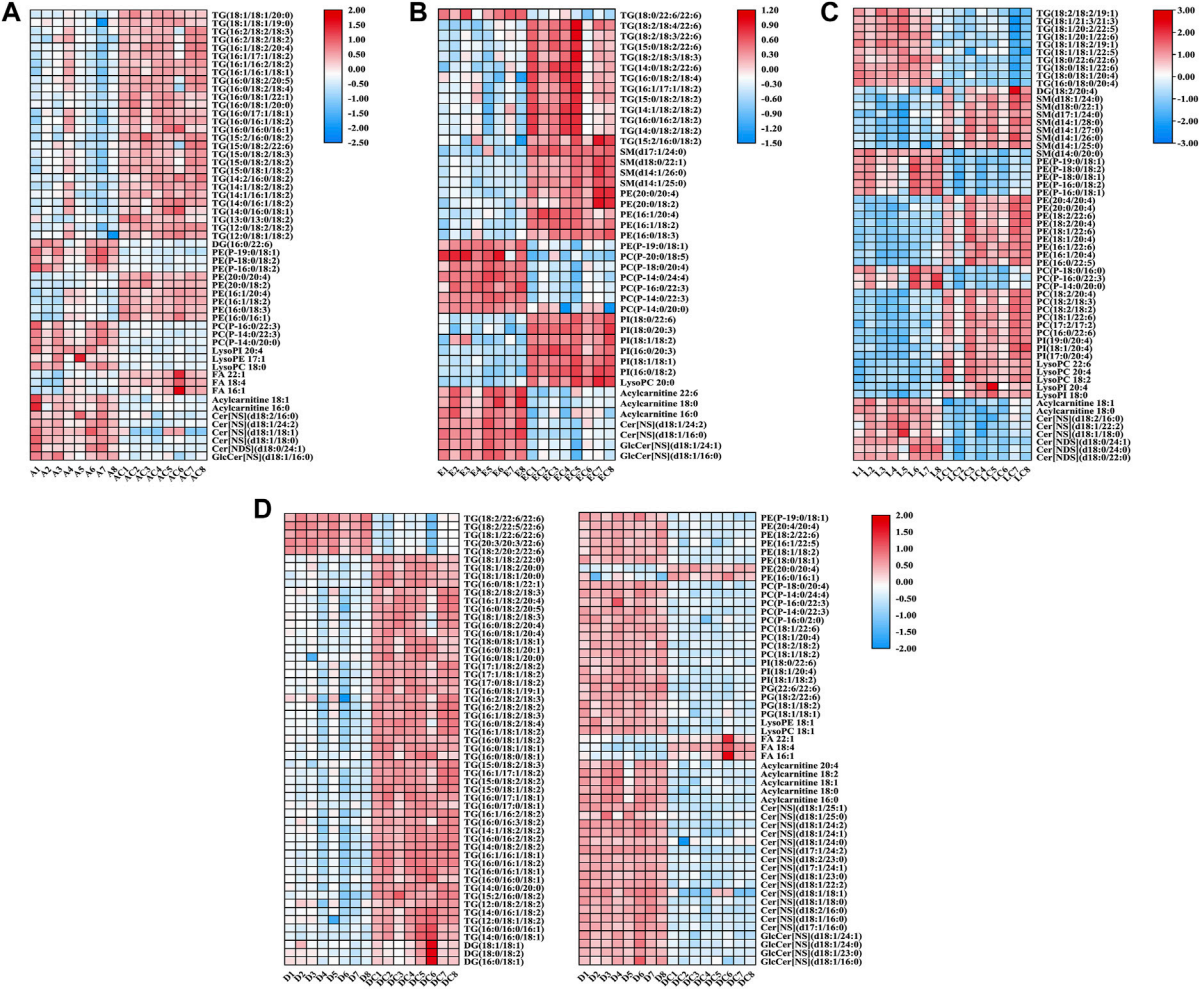
Heatmaps of altered lipid species between acute intrahepatic cholestasis models and NCs. Colors indicated expression quantity, with blue and red representing lower and higher expression, respectively. **(A)** ANIT group vs. ANIT-C group. **(B)** EE group vs. EE-C group. **(C)** LCA group vs. LCA-C group. **(D)** DDC group vs. DDC-C group.

For the 490 detected known lipids, differentially expressed lipids (FC > 2 or <0.5, FDR <0.05) were selected for further analysis. Finally, 55 differential lipids between ANIT and ANIT-C, 110 differential lipids between DDC and DDC-C, 43 differential lipids between EE and EE-C, and 58 differential lipids between LCA and LCA-C were identified (Supplementary Table S1). Heatmaps of altered lipid species between the model group and the NC group were shown in Figure 6. Colors indicated expression quantity of differential lipids in each sample, red represents the increase in concentration and blue represents the decrease in concentration. The darker the color, the stronger the degree of increase/decrease in concentration of differential lipids. As shown in this figure, model groups were significant from NC groups.

**FIGURE 7:**
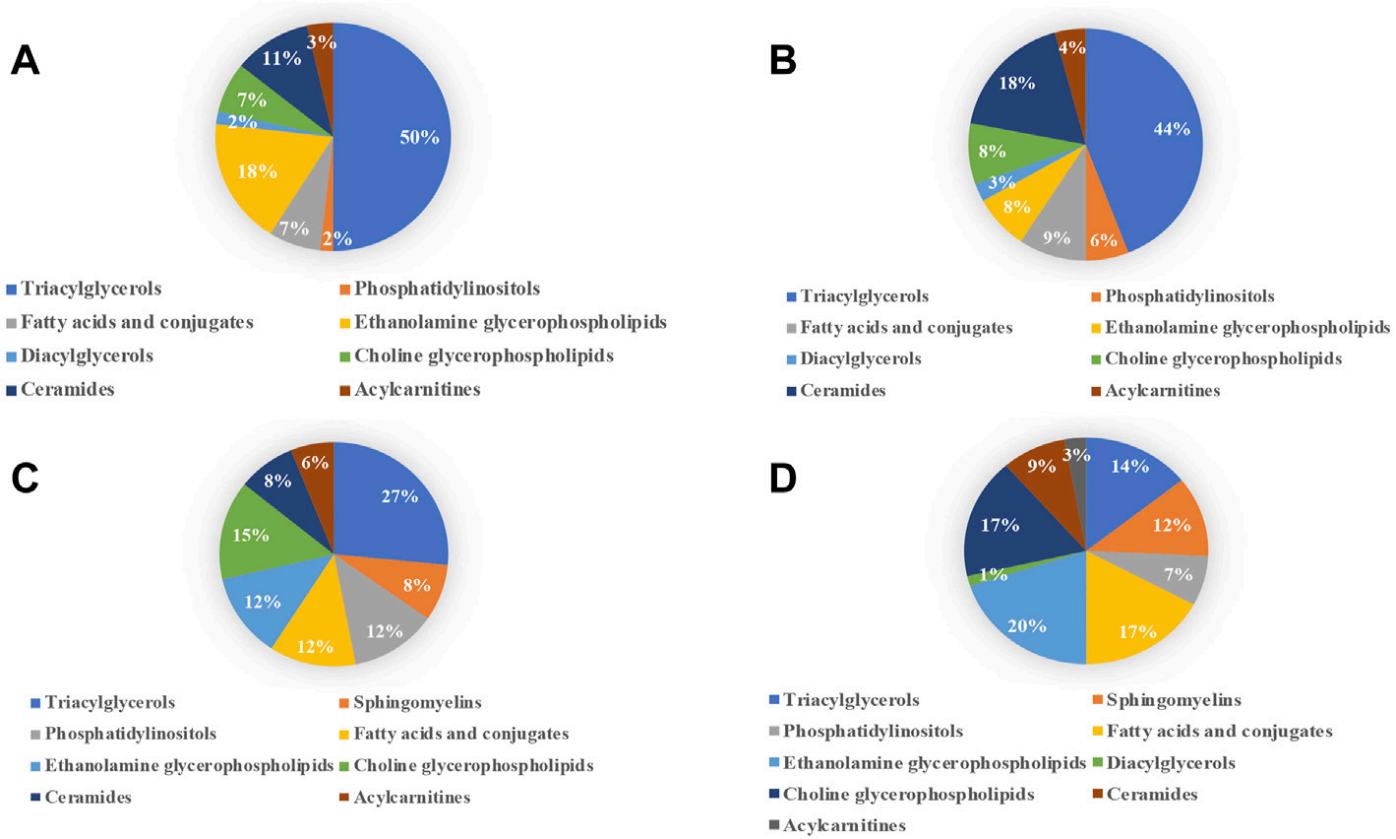
Subgroup classification of lipid disturbances of the four models versus their NC groups. **(A)** ANIT group vs. ANIT-C group. **(B)** DDC group vs. DDC-C group. **(C)** EE group vs. EE-C group. **(D)** LCA group vs. LCA-C group.

Pie diagrams were used to visualize the subgroup classification of differential lipids between models and NCs (Figure 7). As shown in this figure, triacylglycerols and ethanolamine glycerophospholipids were the main differentially lipids in the ANIT group. Triacylglycerols and ceramides were the main differentially lipids in the DDC group. Triacylglycerols and choline glycerophospholipids were the main differentially lipids in the EE group. Ethanolamine glycerophospholipids, fatty acids and choline glycerophospholipids were the main differentially lipids in the LCA group.

### Identification of Differential Small Molecule Metabolites Involved in Each Model

**FIGURE 8:**
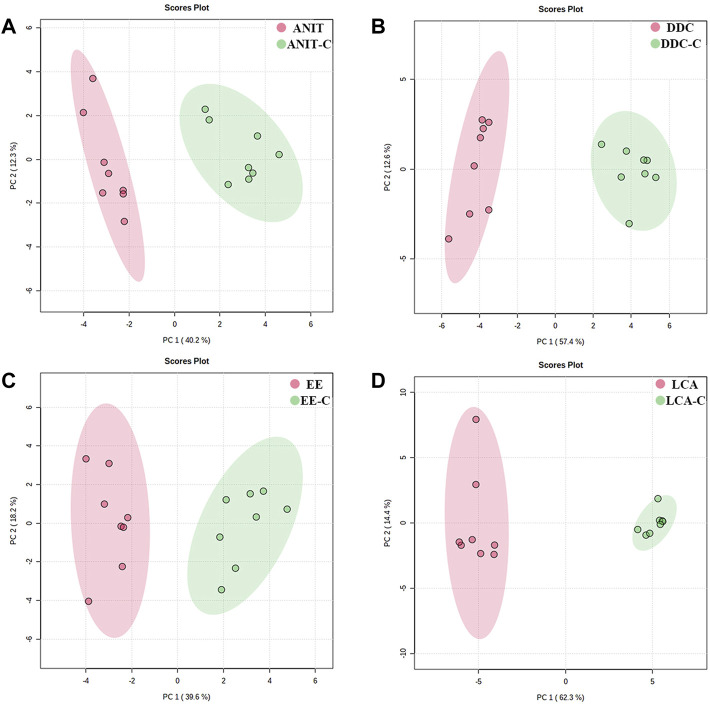
PCA score plots based on the GC-MS profiling of the four models versus their NC groups. **(A)** ANIT group vs. ANIT-C group. **(B)** DDC group vs. DDC-C group. **(C)** EE group vs. EE-C group. **(D)** LCA group vs. LCA-C group. (n = 8).

The metabolomic profiles of liver samples were also investigated by GC-MS. After alignment and normalization of the data sets, we obtained a total of 171 known small molecule metabolites. As shown in Figure 8 and Supplementary Figure S5, the PCA and PLS-DA models showed clear separation between the model groups with their NC groups. The samples in the ANIT group and the DDC group were relatively clustered under GC-MS mode (Supplementary Figure S6), suggesting that the disturbance characteristics of small molecule metabolites in the two models were also similar. The reproducibility of GC-MS data was assessed by using QC samples. As shown in Supplementary Figure S7, the PCA scores plot revealed a clear cluster of the QC samples, indicating the high stability and reproducibility of the instrument.

**FIGURE 9:**
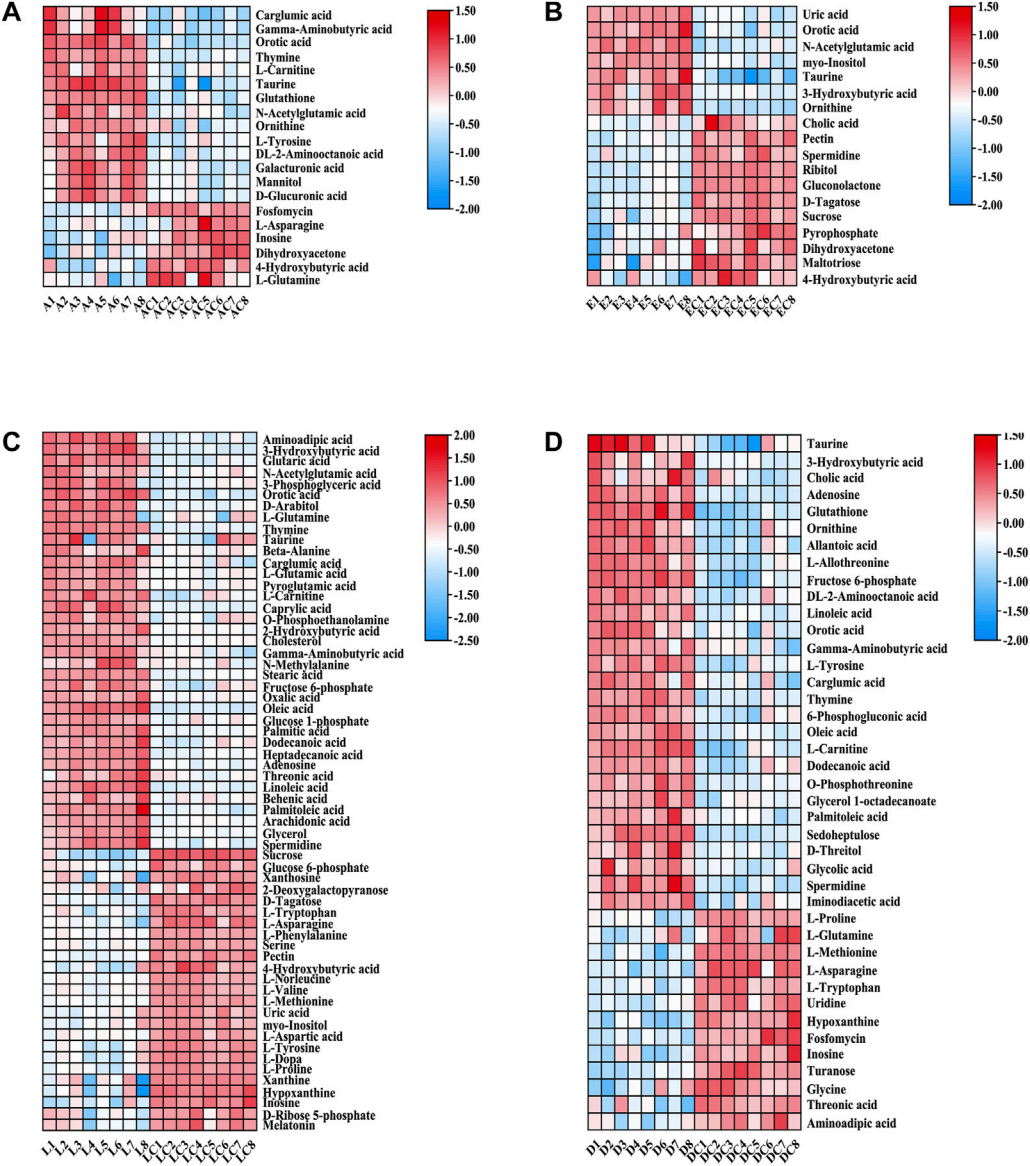
Heatmaps of altered GC-MS metabolites between acute intrahepatic cholestasis models and NCs. Colors indicated expression quantity, with blue and red representing lower and higher expression, respectively. **(A)** ANIT group vs. ANIT-C group. **(B)** EE group vs. EE-C group. **(C)** LCA group vs. LCA-C group. **(D)** DDC group vs. DDC-C group.

For the 171 detected known metabolites, differentially expressed metabolites (FC > 2 or <0.5, FDR <0.05) were selected for further analysis. Finally, 20 differential metabolites between ANIT and ANIT-C, 41 differential metabolites between DDC and DDC-C, 18 differential metabolites between EE and. EE-C, and 62 differential metabolites between LCA and LCA-C were identified (Supplementary Table S2). Heatmaps showed the differential metabolites between the model group and the NC group (Figure 9).

### Pathway Analysis of Differential Lipids and Metabolites

**FIGURE 10:**
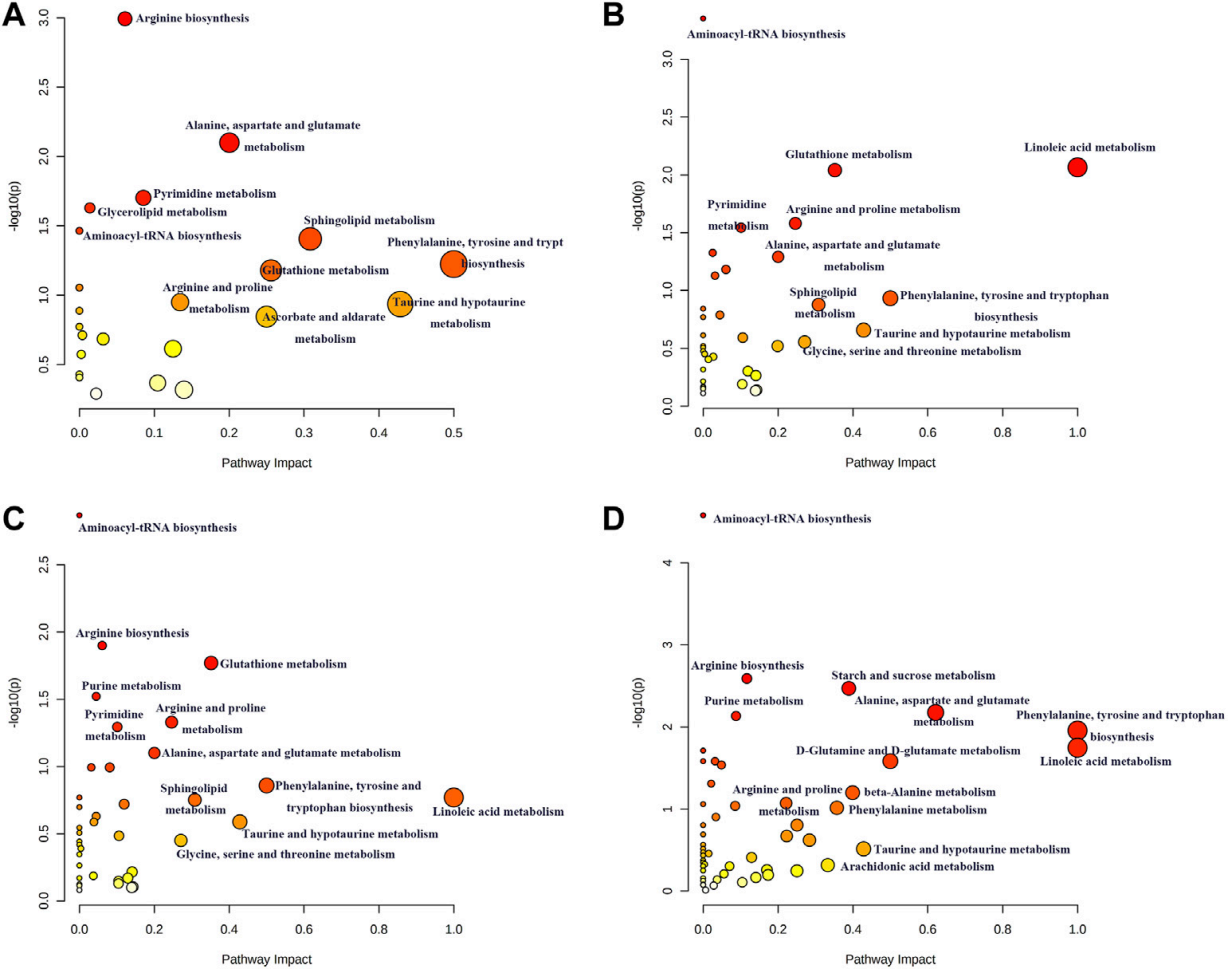
Summary of possible disturbed metabolic pathways involved in ANIT **(A)**, DDC **(B)**, EE **(C)** and LCA **(D)** groups. The vertical coordinate represents -log(*P*) value and the horizontal one represents impact factor. The metabolic pathway with smaller *p* value and larger impact factor is considered more significantly relevant with the mechanism studied.

The disturbed metabolic pathways involved in differential lipids and small molecule metabolites in each model were determined by enrichment analysis using MetaboAnalyst 5.0. As shown in Figure 10, all matched pathways were detected according to pathway impact values of the pathway topology analysis (*X*-axis) and *p* values of the pathway enrichment analysis. The color gradient and circle size indicate the significance of the pathway ranked by *p* values (yellow: higher *p* values and red: lower *p* values) and pathway impact scores (the larger the circle, the higher the impact score), respectively. Significantly affected pathways with a low *p* value and high pathway impact score are identified by name. A series of lipid and amino acid metabolic pathways disorders were involved in the process of acute intrahepatic cholestasis in each model.

## Discussion

**FIGURE 11:**
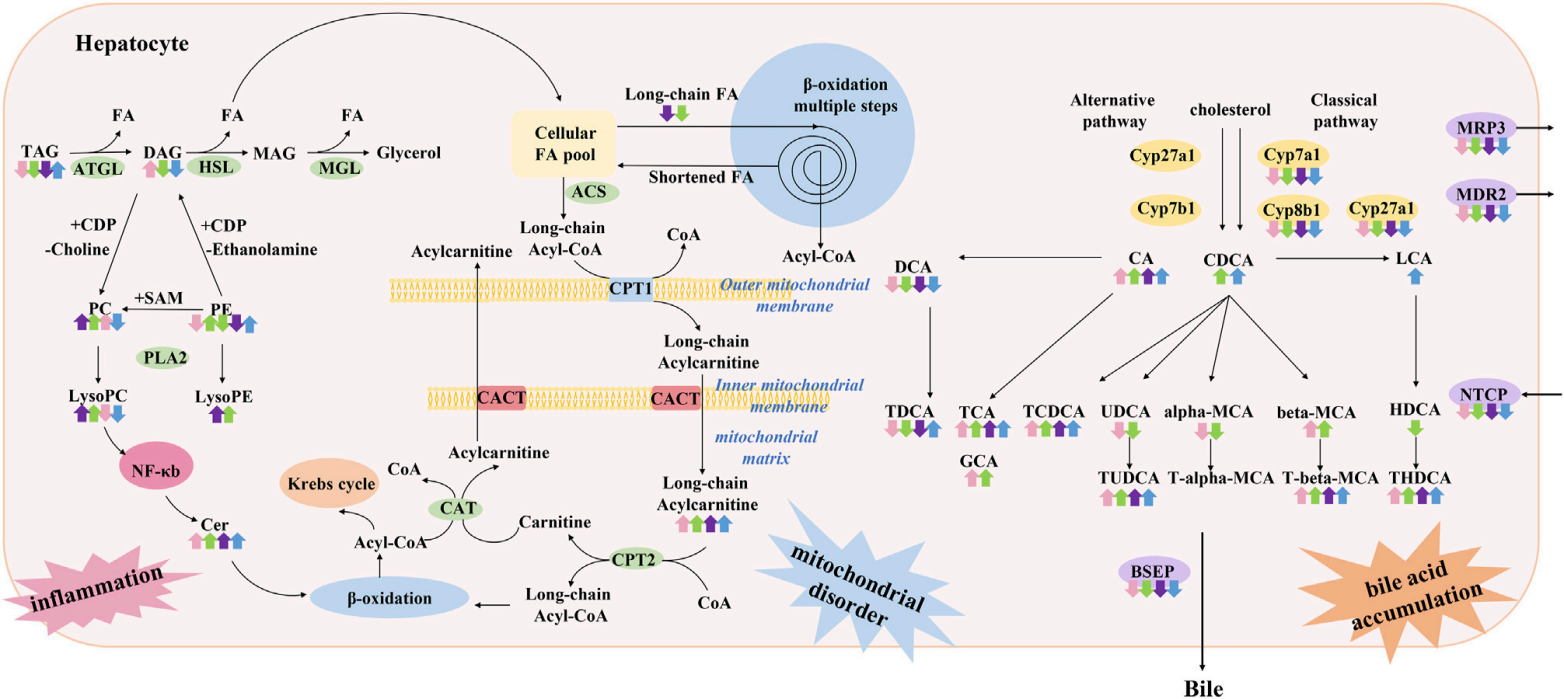
Summary of characteristics of lipid and bile acid metabolism disorders in models of acute cholestatic liver disease in this study. It is speculated that the pathogenesis of this disease is related to hepatocyte inflammation caused by lipid metabolism disorder and toxic bile acid accumulation caused by bile acid metabolism disorder. The four colored arrows indicate the up-regulation or down-regulation of the substance in ANIT (pink arrow) group, DDC (green arrow) group, EE (purple arrow) group, and LCA (blue) group. ATGL: Adipose triglyceride lipase, HSL: Hormone-sensitive lipase, MGL: Monoacylglycerol lipase, PLA2: Phospholipase A2, ACS: Acetyl-CoA synthase, CPT1: Carnitine palmitoyl transferase 1, CPT2: Carnitine palmitoyl transferase 2, CACT: Carnitine acyl transferase.

In this study, we comprehensively analyzed four chemically induced mouse models of acute intrahepatic cholestasis. As shown in Figure 11, we summarized the bile acid disorders and metabolic profile involved in each model group using metabolomics and lipidomic techniques, which also summarized the metabolic pathways involved in each model.

Targeted bile acid level detection results showed that conjugated bile acids of each model group were mainly increased, which was consistent with the clinical manifestations of cholestatic liver disease. Molecular mechanism studies showed that bile acid transporters NTCP, MRP3, MDR2, and BESP were decreased in these models, which would further aggravate the accumulation of bile acids in hepatocytes. In liver disease, the classical and alternative pathways of bile acid synthesis are mutually regulated to maintain the homeostasis of bile acid synthesis under pathological conditions (Jia et al., 2018). In the present study, enzymes related to the classical pathway of bile acid synthesis, such as Cyp7a1, Cyp8b1, and Cyp27a1, were down-regulated, suggesting that the liver tried to maintain bile acid homeostasis by reducing bile acid synthesis under emergency conditions, and shifted bile acid synthesis from the classical pathway to other pathways.

Excessive LCA feeding can induce segmental bile duct obstruction and destructive cholangitis in mice. However, there are various methods to construct acute cholestasis with LCA, such as a 1%LCA diet for 4 days (Fickert et al., 2006) or a 0.6% LCA diet for 7 days (Matsubara et al., 2011). In the present study, a stable acute intrahepatic cholestasis model was constructed by intraperitoneal injection of LCA at 250 mg/kg for 4 consecutive days (Staudinger et al., 2001). In this study, the lipid metabolism profile of the LCA group was different from that of the other groups, mainly manifested by the down-regulation of phosphatidylcholine (PC), lysophosphatidylcholine (LysoPC), and sphingomyelin (SM). Previous studies have reported that LCA induces the disruption of phospholipid/sphingolipid homeostasis through TGF-β signaling, and the decrease of LysoPC and SM levels was related to the elevated lysophosphatidylcholine acyltransferase (LPCAT) and sphingomyelin phosphodiesterase (SMPD) expression (Matsubara et al., 2011). Previous clinical metabolomics studies have shown that LysoPC, PC, and SM levels are down-regulated in PBC and autoimmune hepatitis patients (Lian et al., 2015), which is consistent with the lipid metabolism profile of the LCA group in our study. Besides, it has been reported that in PBC patients, the total primary bile acids (CA and CDCA) were 13.5-fold higher than that of non-cholestatic donors, especially their taurine conjugate (34-fold–46.5-fold accumulation) (Lian et al., 2015). The hepatic level of LCA was also reported to elevate in PBC patients (Fischer et al., 1996). The characteristics of bile acid disorder in PBC patients were more consistent with the LCA group in this study. Therefore, from the perspective of lipid and bile acid metabolism phenotype, LCA-induced cholestasis is more suitable for PBC model than the other three models.

The triglyceride (TG) metabolism disorder in the LCA group was also different from that in the other groups. There were 10 differential TGs in the LCA group, all showing an upward trend. It has been reported that LCA is a Pregnane X receptor (PXR) agonist (Staudinger et al., 2001). PXR is a nuclear receptor and xenobiotic sensor that is highly expressed in intestine and liver. Many studies have demonstrated that PXR activation causes hepatic lipid accumulation in human cell models, and *in vitro* and *in vivo* mouse models (Bitter et al., 2014; Hakkola et al., 2016). This pro-steatotic effect is thought to result from both the activation of lipogenesis and inhibition of β-oxidation (Hakkola et al., 2016) (Chai et al., 2020). PXR is involved in the transcriptional regulation of lipin-1, which participates in TG synthesis (He et al., 2013). PXR also enhances lipogenesis through the induction long-chain free fatty acid elongase (Zhou et al., 2006), and stearoyl-CoA desaturase 1 (Nakamura et al., 2007), both of which are important in fatty acid synthesis. Activation of PXR can also inhibit β-oxidation and dynamically regulate lipid homeostasis. Activation of PXR has a negative impact on carnitine palmitoyl transferase 1A (CPT1A) and 3-hydroxy-3-methylglutarate-CoA synthase 2 (HMGCS2) by repressing the activity of forkhead box protein A2, a regulator of β-oxidation and ketogenesis. CPT1A aids in the transport of long-chain fatty acids into mitochondria for β-oxidation, whereas HMGCS2 is involved in ketogenesis (Nakamura et al., 2007; Nakamura et al., 2014). In conclusion, it was speculated that the up-regulation of TGs in this study was related to LCA’s activating PXR and inhibiting β - oxidation.

Unlike PBC, PSC leads to irregular bile duct obliteration, including formation of multifocal bile duct strictures affecting both intra- and extrahepatic bile ducts, and may finally lead to biliary cirrhosis and liver failure. PSC is frequently associated with inflammatory bowel disease (IBD) and carries a high risk for hepatobiliary and colorectal malignancies. Ideally, immune-genetically predisposed experimental models of PSC would develop fibrous-obstructive cholangitis of both intra- and extrahepatic bile ducts in conditions of gut inflammation, with a marked propensity to develop cholangiocarcinoma over time. Given these prerequisites, unfortunately not fulfilled by a single animal model (Mariotti et al., 2019).

ANIT and DDC are two chemicals commonly used to construct PSC models. However, bile duct phenotypes in ANIT- or DDC-treated rodents resembles the findings in small-rather than large-duct disease/PSC (Fickert et al., 2013). As a hepatotoxic drug, ANIT can lead to impaired expression of bile salt transporters, disruption of hepatic tight junction, and biliary obstruction due to desquamation of the bile duct epithelial cells (Wang et al., 2014; Yang et al., 2017). Chronic DDC feeding can cause progressive biliary tract injury, featuring ductular proliferation, intense pericholangitis associated with onion skin-type periductal fibrosis, which progressed slowly over time, leading to portal-portal bridging, and extending to the large bile ducts (Fickert et al., 2007). Some studies based on time series metabolomics analysis, some studies have explored the changes of metabolites during the progression of DDC-induced chronic cholestatic liver fibrosis model. It was found that changes in TMCA, THDCA, and TCA levels were associated with the progression of DDC-induced cholestatic hepatocyte injury, while the levels of arachidonic acid, proline, leucine and linoleic acid were related to the progression of DDC-induced cholestatic liver fibrosis (Li et al., 2021). In the present study, the ANIT group and the DDC group were clustered together in the lipidomics and metabolomics PCA diagrams (Supplementary Figures S3, S6), suggesting the two groups had similar metabolic profiles.

In this study, TG metabolism was down-regulated in the ANIT and the DDC group. Among them, 28 TGs and one DG were down-regulated in the ANIT group, 47 TGs were down-regulated in the DDC group. The down-regulation of TGs was speculated to be related to hepatocyte injury, and the decline of TG synthesis function in the process of acute cholestasis. In addition, LysoPC (18:0) was significantly increased in the ANIT group, LysoPC (18:1) was significantly increased in the DDC group. These results were consistent with previous reports by (Fang et al., 2017; Wang et al., 2020). *In vitro*, the increase of LysoPC has been shown to activate NF-κB in a concentration-dependent manner (Fang et al., 2017), which may be another mechanism by which ANIT and DDC induce hepatotoxicity. In conclusion, the metabolic characteristics of DDC and ANIT-induced cholestasis models were similar, and we speculated that cholestasis caused by these two chemicals may partially represent small-duct disease/PSC.

Ceramides are members of the sphingolipid family of lipids, and are part of the lipid bilayer structure that constitutes cell membranes (Hannun and Obeid, 2008). Besides, ceramides also have cell-signaling properties. In this study, the up-regulation of ceramide was observed in all model groups. Compared with the NC group, there were 4 up-regulated differential ceramides in the ANIT group, 2 in the EE group, 3 in the LCA group, and 15 in the DDC group. It has been reported that the up-regulation of ceramides may be related to the activation of NF-κB pathway, inducing *de novo* synthesis of ceramides (Zheng RH. et al., 2021). As a key lipotoxic player, ceramide can induce a series of cellular stress responses and eventually lead to cell apoptosis (Summers et al., 2019). In addition, ceramide has a variety of effects on mitochondria, resulting in decreased electron transport chain activity and increased mitochondrial outer membrane permeability. These effects lead to decreased mitochondrial respiration and increased tissue apoptosis (Chaurasia and Summers, 2021). We hypothesized that the up-regulation of ceramides in each model group would result in mitochondrial dysfunction. The accumulation of long-chain acylcarnitine in each model, such as acylcarnitine (16:0) and acylcarnitine (18:1) in the ANIT group, acylcarnitine (16:0), acylcarnitine (18:0), and acylcarnitine (22:6) in the EE group, acylcarnitine (18:0) and acylcarnitine (18:1) in the LCA group, acylcarnitine (16:0), acylcarnitine (18:0), acylcarnitine (18:1), acylcarnitine (18:2), and acylcarnitine (20:4) in the DDC group, also proved the damage of mitochondrial function in each model group (Nguyen and Olzmann, 2017).

Estrogens and their metabolites are involved in pathogenesis of cholestasis diseases, such as intrahepatic cholestasis of pregnancy (ICP), and cholestasis caused by postmenopausal hormone replacement therapy and administration of oral contraceptives (Arrese and Reyes, 2006). EE-induced intrahepatic cholestasis is a rodent model widely used to investigate the molecular mechanisms of estrogen-induced bile secretion dysfunction. In this study, the characteristics of bile acid metabolism in the EE group were similar to those of other models, including the increase of conjugated bile acids and the disruption of bile acid transporters and bile acid synthetic enzymes. This result was consistent with the clinical characteristics of bile acid metabolism in ICP(Cui et al., 2018; Zheng Q. et al., 2021). The liver pathological manifestations of the EE group was significantly different from the other groups. In the EE group, there was no obvious bile infarction in H&E stained liver sections, but inflammatory infiltration was dominant. The metabolic characteristics of the EE group was also different from the other groups. In the lipidomics and metabolomics PCA diagrams (Supplementary Figures S3, S6), the EE group was clearly distinguished from the other three model groups. EE exposure in mice caused a decrease in liver SM levels. There were 4 down-regulated differential SMs in the EE group. The down-regulation of LysoPC (20:0) was also detected in the EE group, which was consistent with the LCA group. TG metabolism was down-regulated in the EE group. A total of 12 TGs were down-regulated in the EE group, which was consistent with the ANIT and the DDC group. Besides, the accumulation of ceramide and acylcarnitine was also detected in the EE group, suggesting that the EE group also had mitochondrial dysfunction.

To conclude, lipidomics and metabolomics techniques were used to summarize the metabolic profiles of the four chemically induced acute intrahepatic cholestasis mouse models in this study. The results showed that bile acid metabolism disorders existed in all model groups, with mainly elevated conjugated bile acids. From the perspective of lipid and bile acid metabolism phenotype, LCA-induced cholestasis was more suitable for characterizing PBC than the other three models. The ANIT group and the DDC group had similar metabolic profiles, which were speculated to be related to the hepatocyte injury caused by the activation of inflammatory pathways. Cholestasis caused by these two chemicals may partially represent small-duct disease/PSC. The metabolic profile of the EE group was different from the other models, suggesting that estrogen-induced cholestasis had its special mechanism. This study will help us to better understand the pathogenesis of acute intrahepatic cholestasis caused by chemicals and provide a reference for the selection of appropriate models for cholestatic liver disease. In future research, we will further explore the relationship between bile acid disorder and lipid metabolism disorder in each model, and deeply explore the cholestasis mechanism of each model.

## Data Availability

The original contributions presented in the study are included in the article/Supplementary Material, further inquiries can be directed to the corresponding authors.
