# Supplementary material for: Integrated Lipidomics and Metabolomics Study of Four Chemically Induced Mouse Models of Acute Intrahepatic Cholestasis
Source: Front Pharmacol. 2022 Jun 8;13:907271. doi: 10.3389/fphar.2022.907271 (PMC9213752; doi:10.3389/fphar.2022.907271)
Supplement: Supplementary file 3 [file DataSheet1.DOCX]

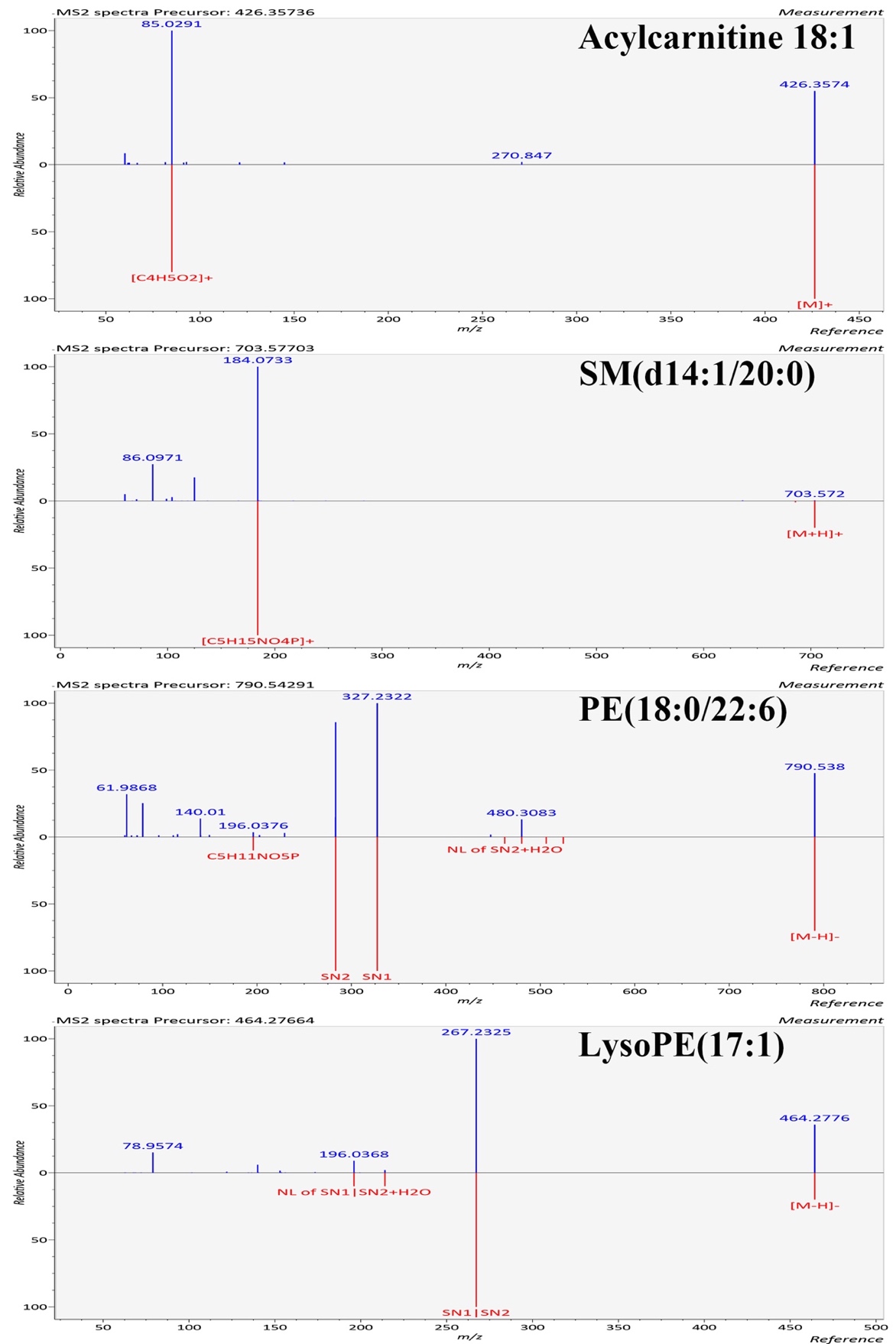
Fig.S1 Selected lipids with matching fragmentation patterns to the LipidBlast library with MS-DIAL software.


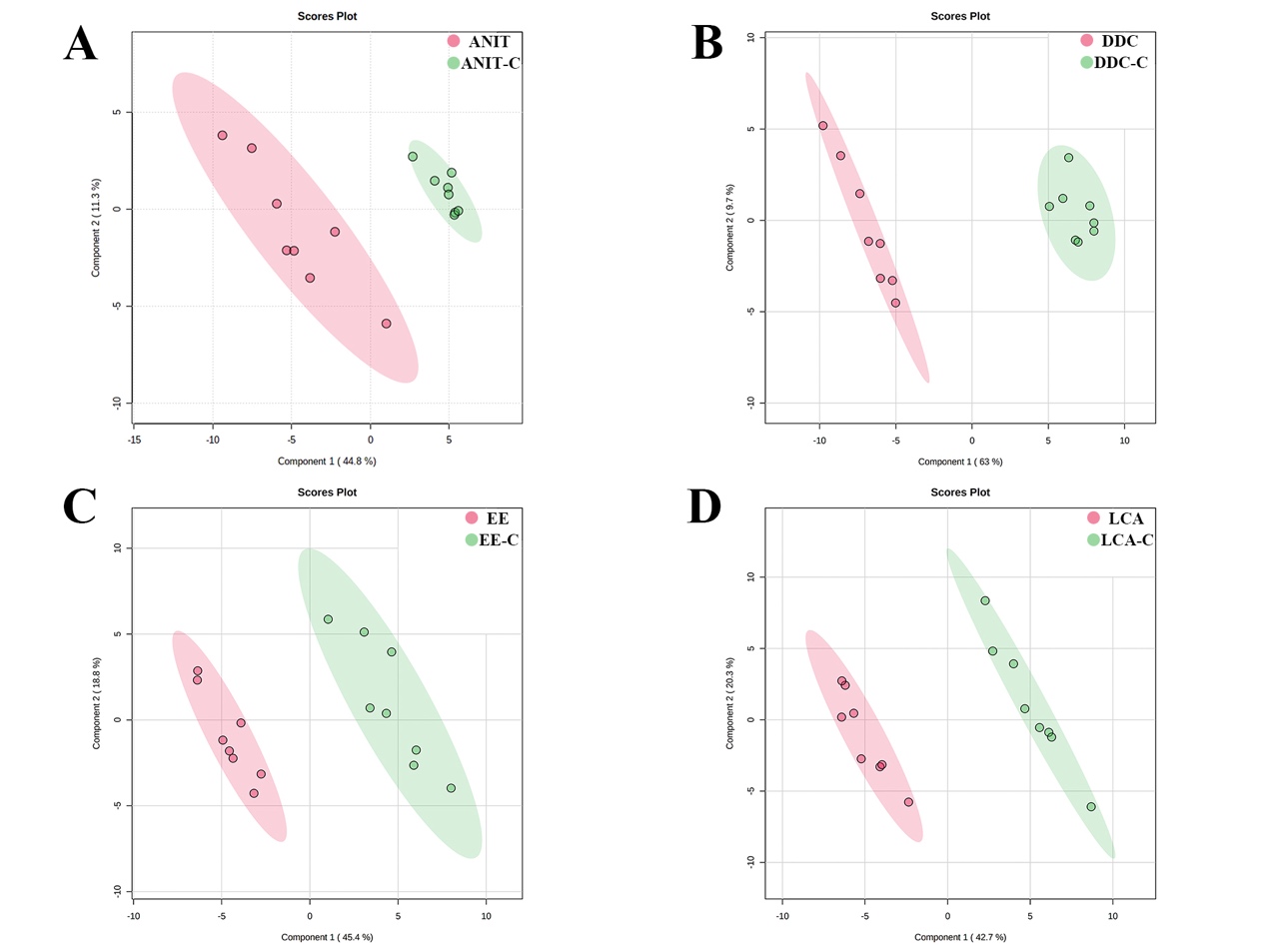
Fig.S2 PLS-DA score plots based on the lipid profiling of the four models versus their NC groups. (A) ANIT group vs. ANIT-C group. (B) DDC group vs. DDC-C group. (C) EE group vs. EE-C group. (D) LCA group vs. LCA-C group. (n = 8).


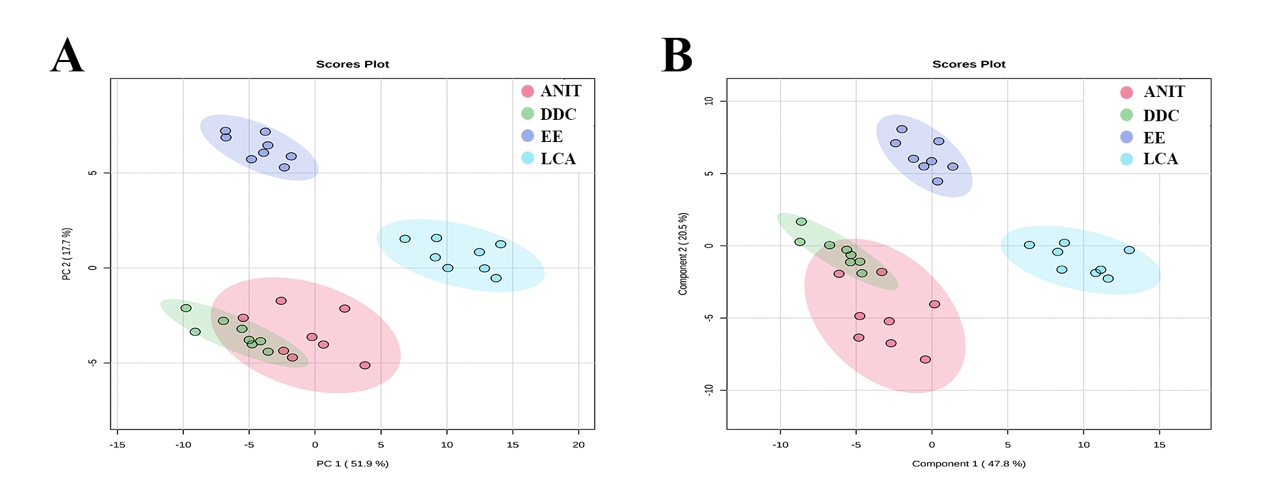
Fig.S3 PCA and PLS-DA score plots based on the lipid profiling of the four models.(A) PCA score plot of liver lipid profiling from the four models. (B) PLS-DA score plot of liver lipid profiling from the four models. (n = 8).


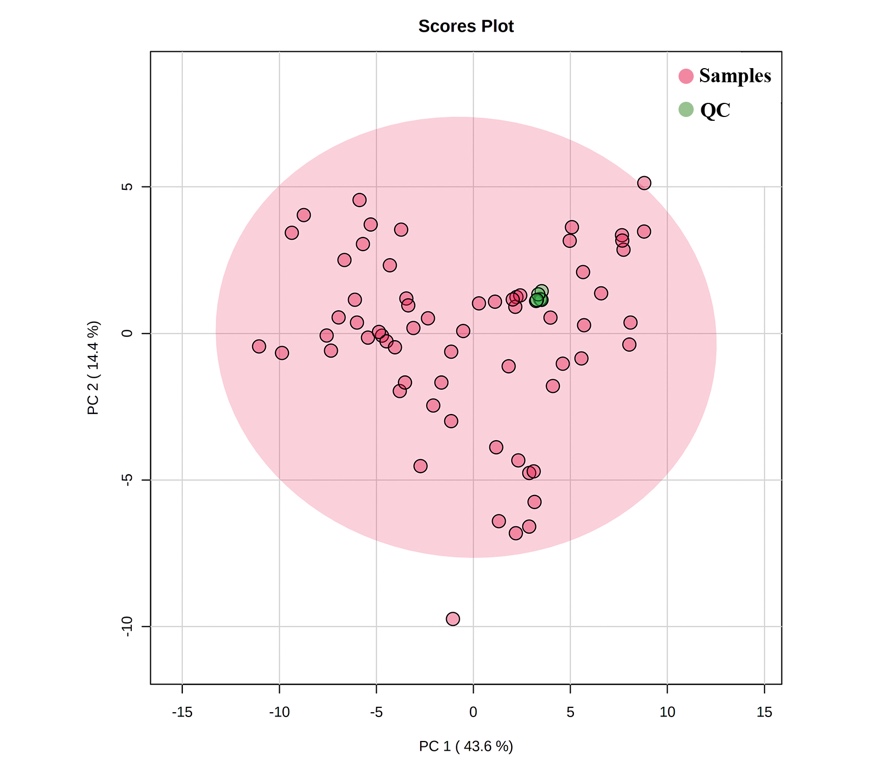
Fig.S4 PCA score plots for QC and the other samples based on the lipid profiling.


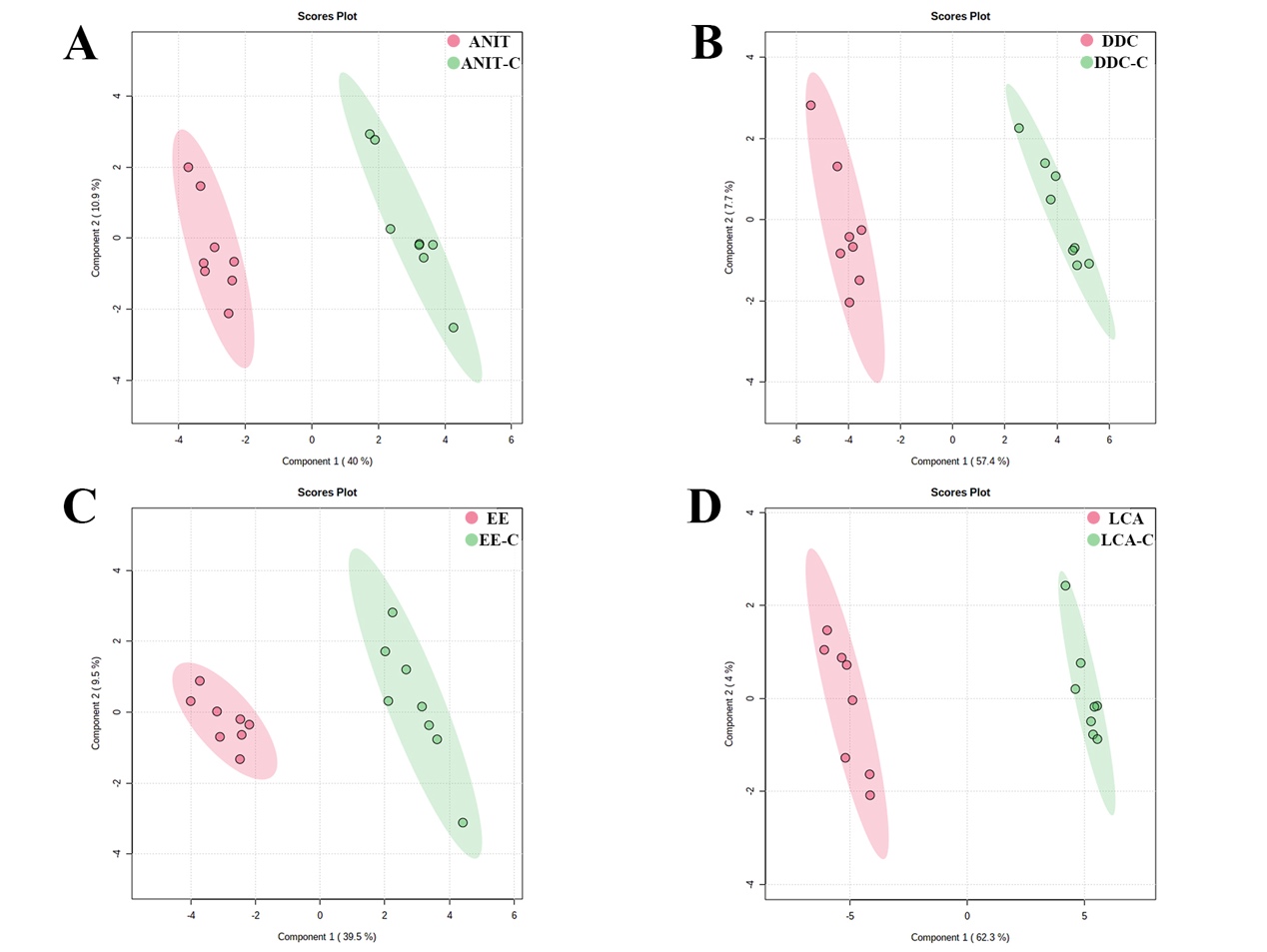
Fig.S5 PLS-DA score plots based on the GC-MS profiling of the four models versus their NC groups. (A) ANIT group vs. ANIT-C group. (B) DDC group vs. DDC-C group. (C) EE group vs. EE-C group. (D) LCA group vs. LCA-C group. (n = 8).


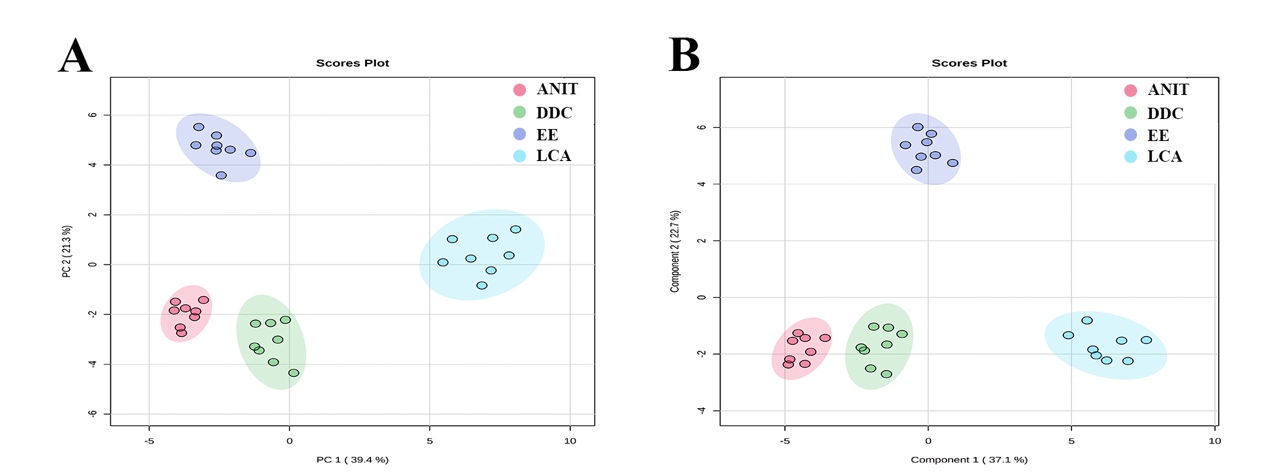
Fig.S6 PCA and PLS-DA score plots based on the GC-MS profiling of the four models. (A) PCA score plot of liver GC-MS profiling from the four models. (B) PLS-DA score plot of liver GC-MS profiling from the four models. (n = 8)


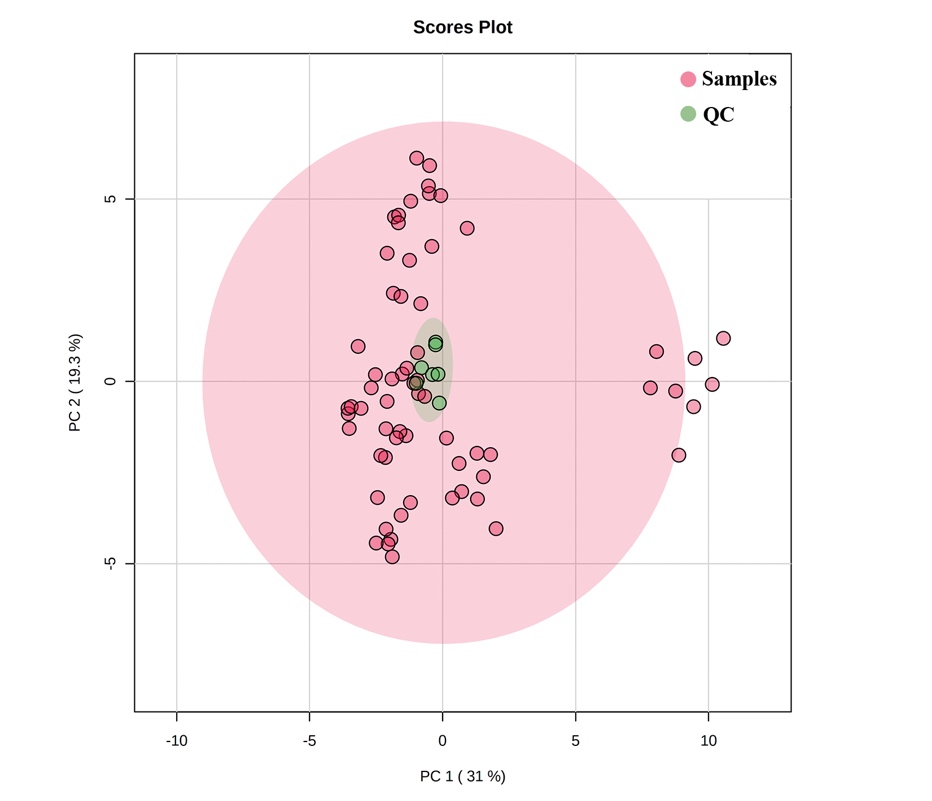
Fig.S7 PCA score plots for QC and the other samples based on the GC-MS profiling.
